# Psychometric Properties and Network Analysis of the Arabic Version of Reinforcement Sensitivity Theory of Personality Scale-Short Version in Patients with Anxiety Disorders

**DOI:** 10.1007/s11126-024-10109-3

**Published:** 2025-01-17

**Authors:** Rasha Mohamed Abdelrahman, Abdulnaser Fakhrou, Mahmoud Ali Moussa, Mohaddeseh Roshan

**Affiliations:** 1 Humanities and social science Research Center(HSSRC), Ajman University, Ajman, UAE; 2 National Center for Examination and Educational Evaluation (NCEEE), Cairo, Egypt; 3 College of Humanities and Sciences, Ajman University, Ajman, United Arab Emirates; 4 Department of Psychological Sciences, College of Education, Qatar University, Doha, Qatar; 5 Educational Psychology, Faculty of Education, Suez Canal University, Ismailia, Egypt; 6 Intelligent Manufacturing Systems (IMS) Centre, Department of Industrial and Manufacturing Systems Engineering, University of Windsor, 401 Sunset Avenue, Windsor, ON N9B 3P4 Canada

**Keywords:** Anxiety disorders, Personality assessment, Psychometrics, Social network analysis

## Abstract

This study aimed to examine the psychometric properties of the Arabic version of a short version of the Reinforcement Sensitivity Theory of Personality Questionnaire (RST-PQ-S) among a sample of 700 patients with anxiety disorders (53.1% were female). Participants completed the RST-PQ-S, NEO-FFI, Positive Mental Health (PMH), and Kessler Psychological Distress scale. Both Exploratory Factor Analysis (EFA) and Confirmatory Factor Analysis (CFA) were employed to test the construct validity of the scale. This study also utilized a network perspective, incorporating Exploratory Graph Analysis (EGA) and centrality measures. As a result of the EFA and CFA, it was determined that the scale consists of 22 items and six subdimensions. These subdimensions were named as follows: “Flight Fight-Freeze System”, “Behavioral Inhibition System”, “Reward Interest”, “Reward Reactivity”, “Goal-Drive Persistence”, and “Impulsivity”. Additionally, the network analysis’s findings confirmed the six-factor structure derived from the construct validity assessment. The results of this study demonstrated that the Arabic version of the personality scale is a valid and reliable tool for assessing personality in Arabic-speaking individuals with anxiety disorders. It has the potential to serve as an important diagnostic instrument in clinical and research settings. These findings may assist psychologists and clinicians in Arabic-speaking countries to better understand how the personality traits and anxiety disorders are related.

## Introduction

One of the main theories in the context of the behavioral sciences is Reinforcement Sensitivity Theory (RST) [1]. In 1970, Jeffrey Gray proposed the Reinforcement Sensitivity Theory (RST) consisting of two primary systems: the first being the Behavioral Approach System, referred to as BAS, and the second being the Behavioral Inhibition System, referred to as BIS [1]. The BAS is believed to govern specific subprocesses related to impulsivity and extraversion [2]. Alternatively, BIS acts as a detector and regulator of conflict. Activation of BIS triggers the identification of conflict, leading to a process of conflict resolution characterized by the emergence of anxiety [3]. This anxiety state serves to influence ongoing psychological processes in a negative direction. Variations in BIS reactivity are thought to underlie individual differences in trait anxiety [4].

### The Revised Reinforcement Sensitivity Theory

The theory underwent updates and revisions (rRST) and has since become widely employed as a neuroscience-based theory of personality. The introduction of the rRST stemmed from several crucial considerations. In its early stages, Gray’s RST predominantly focused on the BIS and the BAS [1, 5, 6]. While foundational, this model lacked the complexity to fully capture the breadth of human motivational and emotional processes [7]. As research progressed, it became apparent that additional systems played significant roles in shaping behavior [1, 6]. In order to tackle this complexity, the rRST introduced three distinct systems: the BAS, the FFFS, and the BIS [8, 9]. The FFFS reacts to conditioned and unconditioned aversive stimuli, often called “repulsor stimuli”. When activated, the responses of the FFFS are influenced by contextual cues and previous learning experiences. Variations in FFFS activity are believed to be associated with individual differences in fear responsiveness [10]. The FFFS system mediates responses to all unpleasant stimuli and elicits defensive behaviors like avoidance, escape, and freezing. It is linked to personality traits associated with fear and clinically connected to phobias and anxiety disorders [11].

Ultimately, the BIS functions as the detector and resolver of goal conflicts, such as FFFS and BAS concurrent activation. It addresses these conflicts by heightening the negative value of stimuli, which may entail activating FFFS until resolution or favoring approach or avoidance behaviors. This expanded perspective provides a more holistic insight into how individuals navigate both approach and avoidance motivations and reactive responses in threatening scenarios. Moreover, by integrating neuroanatomical structures, rRST provides a more grounded understanding of the biological underpinnings of behavior [11, 12]. This adaptation ensures that the theory remains relevant and adaptable to new empirical findings and advances in neuroscience and psychology. It maintains its status as a robust framework for understanding human motivation and behavior.

### Assessment In the Context of the Reinforcement Sensitivity Theory

Currently, various scales are associated with the original RST and its rRST. The BIS/BAS Scale was initially developed based on the theoretical framework of the original RST. Subsequently, as updates were introduced in RST theory, additional scales were designed to reflect these changes [8]. For instance, the Generalized Reward and Punishment Expectancies Scale (GRAPES) evaluates individuals’ general expectations of rewards and punishments across diverse situations. However, this scale has been criticized for inadequate fit indices and its alignment with the original RST rather than rRST.

Another notable tool is the Jackson-5 Scale, which encompasses the five systems described in the rRST [13]. While rRST-based measures exhibit notable strengths, there is an ongoing debate regarding their complete congruence with the revised RST framework [14]. Furthermore, earlier scales, including their fight subscales, often show a stronger positive correlation with the BAS than with the FFFS, raising questions about their construct validity [12].

In response to solving these problems, the RST-Personality Questionnaire (RST-PQ) was originally validated in one study in the United Kingdom (Corr [12]),. In the original version, a six-factor solution was found that included BAS (with four sub-factors: Goal-Drive Persistence, Impulsivity, Reward Reactivity, and Reward Interest), FFFS, and BIS. In the original paper, evidence supporting the validity of the six-factor structure was obtained through convergent and discriminant validity, demonstrated by correlations with existing personality scales. However, this scale was translated and examined regarding psychometric properties in different languages such as Persian [15], Swedish [16], Deutsch [17], and Polish [18]. In summary, the reviewed studies consistently demonstrated that RST-PQ maintains strong internal consistency across various languages, ranging from 0.7 to 0.9, signifying a high level of reliability. Moreover, confirmatory factor analyses conducted across these studies consistently validated the three primary dimensions of the questionnaire. Additionally, the findings strongly revealed significant associations between the RST-PQ and various other personality scales, further supporting its validity and utility in assessing personality traits. Nonetheless, its extensive length makes it time-consuming, which can pose challenges when integrating it into studies with multiple measurement instruments or complex experimental frameworks.

Consequently, its applicability in such contexts is limited. Furthermore, its suitability for large-scale surveys is suboptimal due to space constraints, which is both costly and limited [19, 20]. Additionally, the abundance of items may complicate the assessment of model fit, as lengthy scales may struggle to achieve adequate fit, and the estimation of large models can introduce biases into goodness-of-fit statistics, even with sufficient sample size [21].

A short version of the RST with 22 items derived from the RST-PQ, has been extracted based on theoretical and empirical considerations [22]. These items were chosen from the original study by Vecchione and Corr (2011). The psychometric properties of this shortened version were evaluated, and results showed that this scale has a suitable level of internal consistency. Confirmatory factor analysis, performed on three distinct samples, confirmed the expected six-factor structure, which includes the FFFS, BIS, and four components of the BAS: Reward Interest, Goal-Drive Persistence, Reward Reactivity, and Impulsivity. While the internal consistency for the six factors was generally satisfactory, the omega coefficients for the FFFS, reward reactivity, and impulsivity scales were below optimal standards, ranging from 0.60 to 0.70.

### The Interplay Between Reinforcement Sensitivity Theory and the Big Five Personality Traits

Conceptualizing personality as the interplay between biological systems and observable traits offers a rich framework for understanding human behavior. The RST provides a neurobiological lens through which we can examine the roots of personality, positing that our behavioral responses are governed by systems such as the BIS, BAS, and FFFS. These systems do not operate in isolation but interact closely with the Big Five personality traits. For several reasons, understanding the association between RST dimensions and the Big Five personality traits is crucial. First, it allows for a more comprehensive understanding of personality by linking observable behaviors to underlying neurobiological processes. The Big Five traits—Neuroticism, Extraversion, Openness, Agreeableness, and Conscientiousness—are well-established in psychology as broad descriptors of individual differences. However, they do not explain why individuals exhibit certain patterns of behavior. RST fills this gap by providing a mechanistic explanation rooted in neurobiology, explaining how different brain systems underlie these traits and drive specific behaviors [15].

Second, examining the associations between RST and the Big Five enhances the predictive power of personality assessments. For example, by understanding that high BIS activity corresponds with high Neuroticism, researchers and clinicians can better predict an individual’s susceptibility to anxiety and emotional disorders. Similarly, knowing that BAS activity correlates with extraversion can help predict how a person might respond to reward-driven scenarios, social interactions, or motivational interventions. This linkage is particularly useful in applied settings, such as clinical psychology, where understanding the root causes of personality traits can inform treatment strategies [15, 23].

### Using The Network Perspective: Why and How?

Besides the promising results of the RST-PQ-S, the scale development process was faced with an important methodological limitation. The authors examined the psychometric properties of RST-PQ-S using a traditional approach. However, in behavioral sciences, variables are often conceptualized under the assumption that traits are latent variables (constructs) that cannot be directly observed but rather influence observable behaviors [24, 25, 26]. This distinction is crucial because latent variables represent the underlying psychological traits that explain the variance in observed behaviors. In the absence of factors specific to behavior, these latent constructs help elucidate a significant portion of the variability and covariation among observed behaviors. For instance, when examining personality traits like extraversion or intelligence, researchers expect individuals who exhibit tendencies of extroversion or higher intelligence to score higher on assessments measuring these constructs. This is a reflection of what is known as “reflective measurement models,” where the observed behavior (e.g., scores on a personality test) reflects the individual’s standing on a particular latent trait. This raises important questions about the quantitative structure inherent to psychological constructs, including their independence from specific measurement instruments and the impact of construct variations on measurement outcomes. Therefore, while traditional psychometric methods (such as classical test theory or factor analysis) have been widely used, they often fail to account for the complex and dynamic relationships between these latent traits and the observed behaviors. Network analysis, such as the use of EGA, offers a more sophisticated and nuanced way of examining these interrelations, allowing for a more accurate representation of how individual traits, such as those measured by the RST-PQ-S, interact and contribute to the broader psychological profile of an individual.

By shifting the focus to latent trait models and network analysis, we can uncover deeper insights into the structure and functioning of psychological constructs, which would otherwise remain obscured by traditional measurement methods. This methodological approach holds great promise for advancing the field of psychometrics and improving the accuracy and relevance of personality assessments like the RST-PQ-S.

Network analysis is a novel approach in behavioral sciences, especially psychology. This model has proposed new features for evaluating correlations among variables (such as symptoms, values of psychiatric disorders, and items of scales) in the entire network, including positions, structure, shape of ties, and dyadic properties [27]. An important and novel branch of network analysis is EGA. EGA offers several advantages over traditional techniques such as Exploratory Factor Analysis [28] and CFA when dealing with complex datasets, particularly in the field of network analysis [29, 26]. Unlike traditional statistical methods that impose specific assumptions about the underlying data structure, EGA allows researchers to analyze variables’ relationships flexibly and data-driven [30]. In EGA, the network is represented as a graph, where nodes represent individual variables or entities, and edges represent their connections or relationships. By analyzing the graph structure, researchers can identify key nodes, clusters, and connectivity patterns within the network. Furthermore, this perspective is able to uncover hidden structures or emergent properties within the data that traditional statistical methods may not capture. By visualizing the network and exploring its properties, researchers can gain insights into the underlying structure and organization of the data [31, 32].

### Reinforcement Sensitivity Theory of Personality In Anxiety Disorders

There is a new trend in evaluating the psychometric properties of personality scales among clinical samples.For example, one study evaluated the psychometric properties of the 20-item Toronto Alexithymia Scale (TAS-20) among a sample of patients with personality disorders [33].In the context of the RST-PQ, a study used confirmatory factor analysis and exploratory structural equation modeling to examine its factor structure and its relationship with ADHD symptoms. They found that the ESEM model, which identified six factors, provided better support than the CFA model. Both inattention (IA) and hyperactivity/impulsivity (HI) symptom groups were positively associated with the RST-PQ constructs of the BIS and BAS Impulsivity. Additionally, IA symptoms were negatively associated with the BAS-Goal-Drive Persistence.

One clinical population in which personality plays an important role in their disorder presentations is patients with anxiety disorder. For example, one study showed that HEXACO personality traits play a significant role in mediating the relationship between fear of negative evaluation and social interaction anxiety [34]. Another study found that there is a significant relationship between personality traits and emotional dimensions, which may contribute to the development of emotional disorders such as anxiety and depression. The study reviewed the evolution of personality theories and dimensional emotional theory, highlighting the overlap between emotional and personality dimensions. Results indicated that extroversion and introversion were linked to hedonic dimensions, encompassing happiness and sadness, while neurotic dimensions were associated with emotional arousal. Additionally, basic emotions like disgust, anger, and fear were linked to certain personality traits, such as self-centeredness and instability [35]. So, it’s necessary to evaluate the psychometric properties of RST-PQ among clinical samples, especially among anxiety disorders.

### Present Study

The application of the RST-PQ-S in the Arabic-speaking population has faced obstacles due to the lack of evidence-based validation in the Arabic language. Utilizing the RST-PQ-S questionnaire is particularly significant for the Arab community, as it can enhance knowledge and comprehension within this domain. This questionnaire enables the analysis of behavioral and psychological traits specific to Arab society, with its findings aiding in advancing theories and models about this demographic. Using the RST-PQ-S, researchers can identify optimal methods and strategies to support personality development and behavioral regulation among the Arabic-speaking community. Furthermore, this initiative is pivotal in broadening the existing literature on behavioral psychology and understanding the influence of various factors on their behaviors. Given the importance and advantages of this questionnaire, the validation of a verified Arabic version of this tool becomes imperative. In this study, the NEO-FFI questionnaire was used to evaluate the convergent validity of the RSTPQ questionnaire due to its scientific and cultural relevance. The NEO-FFI is a well-regarded instrument for assessing the five major personality traits (Neuroticism, Extraversion, Openness, Agreeableness, and Conscientiousness) and has a long history of use in international research. It thoroughly covers essential traits such as neuroticism, agreeableness, conscientiousness, openness, and extraversion, which align with the study’s goals in assessing convergent validity. Additionally, the NEO-FFI has been translated into Arabic and culturally adapted for use in Iran, ensuring appropriate linguistic and cultural relevance for this study. The RST-PQ questionnaire is also important for the Arab community and can enhance understanding and knowledge in this area. Recognizing the gaps in current literature and the need for culturally tailored assessment instruments, this study investigated the psychometric properties of the Arabic adaptation of the RST-PQ-S. Our specific objectives were to:

*Validate the Factorial Structure of the RST-PQ-S in the Arabic Context with Anxiety Disorders*

*Translation and Cultural Adaption of RST-PQ-S Into the Arabic Language*

*Assess the Convergent Validity of the RST-PQ-S by Examining its Correlations with Established Personality Measures*

*Evaluate the Factor Dimension (EGA) of the RST-PQ-S*

### Determine the Reliability of the Arabic RST-PQ-S Through Internal Consistency and Stability Analyses

By fulfilling these aims, this study has enhanced our understanding of reinforcement sensitivity within the Arab community, thereby increasing the applicability of the RST-PQ-S in both cross-cultural research and clinical contexts. To assess convergent validity alongside the RST-PQ-S questionnaire, we incorporated the NEO-FFI questionnaire, recognizing its scientific and socio-cultural significance. The NEO-FFI is renowned for its comprehensive evaluation of the five major personality factors: neuroticism, extraversion, openness, agreeableness, and conscientiousness, and has a robust history of global research. This instrument thoroughly encompasses key constructs such as neuroticism, agreeableness, conscientiousness, openness, and extraversion, all pertinent to our study’s objectives regarding convergent validity. Moreover, the translation of the NEO-FFI into Arabic and its cultural adaptation to the Arab-speaking community ensures the necessary linguistic and cultural alignment for our study’s purposes.

## Method

### Participants

This cross-sectional study was conducted between March and July 2023 in several Arab-speaking countries: Bahrain, Qatar, Oman, and Kuwait. A convenience sample sampling method was used for data gathering. Inclusion criteria were: agreement to written informed consent, being an adult (above 18 years), completing the questionnaires, and being able to read and speak Arabic.

### Procedure

A detailed notice was carefully prepared and published online throughout Bahrain, Qatar, Oman, and Kuwait to facilitate participation in this research project. This announcement comprehensively outlined the steps for joining the study, its objectives, assurances of confidentiality, and other pertinent details. The recruitment strategy involved the development of an electronic questionnaire utilizing the Google platform. The questionnaire was promoted on popular social media platforms, including Telegram and Instagram, where participants could conveniently access the survey through the links provided. To identify participants with anxiety disorders, all individuals completed the Arabic version of the Beck Anxiety Inventory (BAI). Those who scored above the suggested cutoff of 16 were classified as having an anxiety disorder and were subsequently invited to complete the study scales [36].

The initial landing page of the questionnaire provided comprehensive information regarding the study’s nature and objectives and assurances regarding data privacy. Participants who consented to participate in the study completed the designated scales. To mitigate the possibility of participants submitting the online questionnaire multiple times, several precautions were implemented to maintain control over such issues. First, IP tracking was employed to identify and block multiple submissions from the same device or network. Additionally, clear instructions were included at the outset of the questionnaire, underscoring the importance of providing accurate and truthful responses explicitly warning that duplicate submissions would be detected and excluded from the analysis.

### Measures

NEO-FFI stands as a widely recognized self-report scale assessing the Big Five personality traits: extraversion, neuroticism, agreeableness, openness to new experiences, and conscientiousness. Comprising 60 items, with twelve items allocated to each personality trait, this scale has been extensively utilized across diverse linguistic and cultural contexts worldwide. In line with this, the Arabic version of the NEO-FFI underwent evaluation by Alansari in 1997 Their findings unveiled satisfactory reliability for the conscientiousness and neuroticism subscales, boasting values of 0.83 and 0.80, respectively. Conversely, the agreeableness and extraversion subscales demonstrated only moderate reliability, recording values of 0.60 and 0.58, respectively. However, the subscale measuring openness to new experiences failed to demonstrate significant internal consistency, as indicated by a Cronbach’s alpha of 0.39 [37].

***The short version of the reinforcement sensitivity theory of personality questionnaire (RST-PQ-S)*** is a 22-item self-report scale developed by Vecchione and Corr, designed to assess the BIS, BAS, and FFFS subscales, as previously mentioned and detailed in the introduction. In the initial study, CFA results were validated in a separate sample, and construct validity was established through correlations with BIS/BAS scales. Additionally, test-retest correlations demonstrated satisfactory to good levels of temporal consistency over a four-week period. The findings revealed a considerable overlap with the original full-length RST-PQ, indicating the comparability of the two versions. Latent State-Trait analyses indicated that the items of the RST-PQ-S predominantly capture stable interindividual differences across various situations. These results suggest that the RST-PQ-S offers an efficient, valid, and reliable alternative to the lengthier RST-PQ. One of the main aims of the current study was to evaluate the psychometric properties of the Arabic version of this scale.

***PMH scale*** was employed to assess the psychological and subjective dimensions of well-being. This tool comprises nine items, each rated on a 4-point Likert scale from 0 (strongly disagree) to 3 (strongly agree), with higher total scores indicating greater levels of positive mental health. Convergent validity was established, as evidenced by a moderate correlation coefficient of − 0.65 between the Saudi Arabian version of the PMH scale and the Arabic version of the Beck Depression Inventory-II (BDI-II). Additionally, in the Arabian version, each item demonstrated a good correlation with the overall scale, with correlation coefficients ranging from 0.42 to 0.67 (Almubaddel [38],.

***Kessler Psychological Distress Scale*** measures non-specific psychological distress across behavioral, emotional, cognitive, and psychophysiological dimensions. The scale was developed with susceptible items designed to detect severe psychological distress in the general population (Easton, S.D,. et al.2017). The ten-item version (K10) assesses how frequently respondents experienced symptoms such as nervousness, hopelessness, sadness, worthlessness, and fatigue over the past month. Responses are recorded on a 5-point Likert scale, ranging from 1 (none of the time) to 5 (all of the time), with the total score (ranging from 10 to 50) indicating the level of psychological distress—higher scores reflect greater distress. Research has indicated that a score of 24 is the optimal threshold for identifying psychological disorders. Reliability analysis of the Arabic version of the scale showed satisfactory results for both the K6 and K10 versions, with Cronbach’s alpha coefficients of 0.81 and 0.88, respectively [39].

### Translation and Cross-Cultural Adaptation Process

The original version of the RST-PQ-S, which served as the basis for our study, comprised 22 items drawn from the full version of the RSTPQ, encompassing items related to all BAS, BIS, and FFFS factors. This process yielded a condensed version with adequate items for each factor, ensuring a comparable length to other tools designed for evaluating similar constructs. We translated and cross-culturally adapted the RST-PQ-S in different stages according to diffrent well-stablished papers [40, 41]. Initially, two independent translators translated the original version of RST-PQ-S into Arabic. One was a clinical psychologist proficient in English, while the other was an English teacher with over ten years of experience in English translation and literature. They produced forms “I” and “II”. In the second stage, the mentioned translators merged their forms and produced a final version called “III.” Subsequently, two translators from Canada and America, proficient in Arabic but lacking training in psychology and access to RST-PQ or similar measures, translated the synthesized Arabic version “III” back into English. The final versions, “IV” and “V,” were generated without referencing the original survey. In the fourth stage, they merged their files called “VI.” In the fifth stage, all generated files were compared by an expert committee, including the authors of the current paper, in order to modify the final version and produce the ultimate version for use, called “VII.“. In terms of challenges, one notable issue we encountered was the translation of certain psychological terms that did not have direct equivalents in Arabic. For instance, terms like “behavioral inhibition” and “behavioral activation” required careful consideration to ensure that the intended meaning was maintained. Additionally, there were challenges in adapting the scale to capture cultural differences in emotional expression and social behavior. However, these challenges were addressed through consultation with experts in psychology and cross-cultural research to ensure that the translated scale was psychometrically sound and culturally appropriate.

### Pilot Study

In a preliminary investigation, thirty psychology students evaluated the clarity and comprehensibility of the Arabic version (VII). They were asked to utilize the tool and note any ambiguous items in their responses. Following this, clinical psychologists and psychiatrists with doctorate degrees reviewed and endorsed the tool based on feedback. Subsequent adjustments to the Arabic version were made, including the incorporation of a Likert scale with response choices spanning from “not very much” (1) to “very much” (6), as deemed necessary.

### Data Analysis

This investigation employed EFA to derive factors from the RST-PQ-S. Sample adequacy was assessed using the Kaiser-Meyer-Olkin (KMO) test, while Bartlett’s Sphericity Test was utilized to examine the sphericity assumption. KMO values falling within the range of 0.7 to 0.8 were deemed satisfactory, while values between 0.8 and 0.9 were considered excellent. Subsequently, latent factors were extracted using Principal Axis Factoring (PAF) with Promax rotation, guided by examination of a scree plot [42, 43]. CFA was subsequently conducted, and model evaluation was performed using the ML estimator in AMOS software. Goodness-of-fit indices including chi-square test/degrees of freedom (χ2/df), Adjusted Goodness of Fit Index (AGFI), Goodness of Fit Index (GFI), Comparative Fit Index (CFI) [44], Standardized Root Mean Square Residual (SRMR), root mean square error of approximation (RMSEA), and Akaike Information Criterion (AIC) were utilized for model assessment. Threshold values indicating acceptable fit were established: CFI, GFI, AGFI > 0.90; χ2/df < 3; and RMSEA, SRMR < 0.08 (M. Kordbagheri et al., [15]. Chi-square difference was employed for model comparison [41, 45, 46]. Modification indices exceeding 30 were considered for model refinement. Pearson correlation analysis was additionally conducted to explore the relationship between RST-PQ-S and NEO-FFI scales.

EGA was conducted using the Desktop version of R-software (version 4.2.1, “Funny-Looking Kid”). EGA integrates a network estimation algorithm with a community detection algorithm. The network estimation employs a Gaussian graphical model, implemented via the qgraph package, and incorporates an extended Bayesian information criterion model selection method alongside the Least Absolute Shrinkage and Selection Operator (LASSO) regularization procedure [30, 47]. LASSO uses the correlation matrix of observable variables to produce a sparse inverse covariance matrix. After computing the partial correlation matrix, the Walktrap algorithm in the igraph package is used to determine the number of dense subgraphs [48]. Walktrap utilizes “random walks” between nodes, assigning higher probabilities to transitions between proximal nodes, resulting in node modules with high connectivity [30, 47].

## Results

### Descriptive Statistics

From the initially selected population-based sample, 809 individuals were examined to assess their eligibility for data analysis. Of these, 63 participants were excluded due to incomplete responses to the RST-PQ-S questionnaire. Additionally, 27 participants were excluded for abnormally short response times, which were defined as completing the questionnaire in under 5 min, suggesting potential random answering. Moreover, duplicate responses from the same participants, identified by repeated IP addresses, excluded 14 responses. Consequently, 700 participants were included in the final data analysis set.

In this study, 372 participants (53,1%) were female, and the others were male. Regarding education level, 301 individuals (43%) held a diploma or sub-diploma, 259 participants (37%) possessed a bachelor’s degree, and 140 respondents (20%) had a master’s degree or higher. Additionally, concerning occupation, 245 participants (35%) were housewives, 84 individuals (12%) were employees, and 371 people (53%) were self-employed. The skewness and kurtosis values for all items fell within the range of -0.73 to 0.46 and − 0.91 to -0.22, respectively, confirming the assumption of univariate normality. Additionally, all items’ mean and standard deviation (SD) values ranged from 1.46 to 3.29 and 0.57 to 1.10, respectively. All samples were randomly divided into independent subgroups: Subgroup 1 was allocated for evaluating CFA (350 participants), while the subsequent subgroup was designated for evaluating EFA (350 participants). Additionally, for GA, which aligns with the dimensional approach of behavioral sciences, the analysis was conducted on 700 participants.

### Exploratory Factor Analysis

**Fig. 1:**
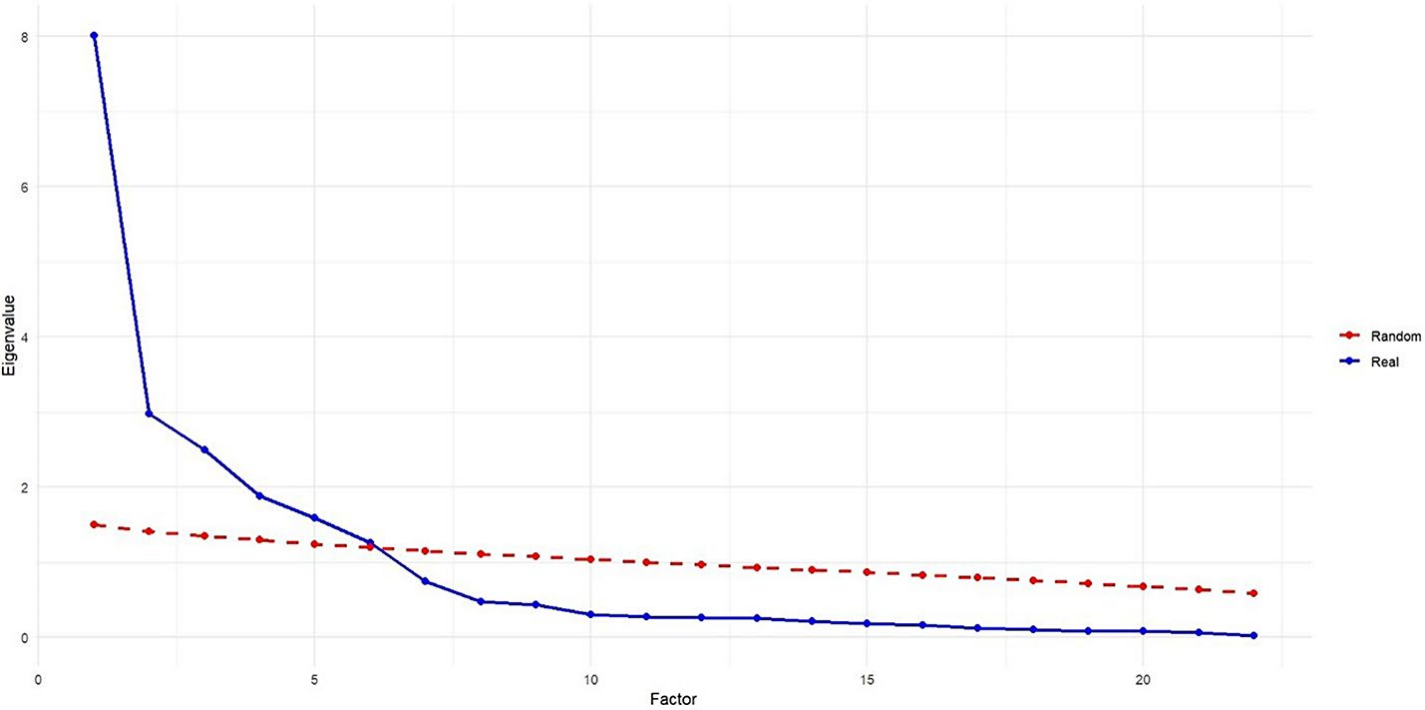
Comparison of Eigenvalues for Real and Random Data in Parallel Analysis

The Kaiser-Meyer-Olkin (KMO) sampling adequacy index, a metric utilized to gauge the appropriateness of data for exploratory factor analysis, returned a robust value of 0.841. This score underscores the suitability of the dataset for the intended analytical approach. Furthermore, Bartlett’s test, yielding a statistically significant outcome of 7084.46 with a significance level of *P* < 0.001, corroborated the dataset’s aptness for factor analysis, bolstering confidence in the subsequent analyses. Upon delving into the factor structure of the RST-PQ-S, the examination identified six factors, each possessing eigenvalues surpassing one, as corroborated by the scree plot (Fig. 1-blue line). This observation signifies a substantial contribution of these factors to the overall variance of the RST-PQ-S structure. Employing Promax rotation, the analysis uncovered that these six factors collectively accounted for 72.5% of the variance within the RST-PQ-S structure, as depicted in Table 1. This comprehensive analysis sheds light on the underlying factors shaping the RST-PQ-S construct, providing valuable insights into its dimensional structure and contributing significantly to our understanding of reinforcement sensitivity.

**Table 1:** Exploratory factor analysis for RST-PQ-S

Thematic Facets	EFA								
	FFFS	BIS	RI	GDP		RR		IMP	
19. If fire alarms were to sound in a shopping mall, I would promptly evacuate.	**0.94**	0.28	0.07	0.09		0.02		0.20	
39. I would immediately become motionless if I opened the door to discover a stranger in the house.	**0.94**	0.14	0.06	0.19		0.07		0.19	
46. If I witnessed a group of dogs running around and barking at people in the park, I would promptly depart from the area.	**0.75**	0.06	0.012	0.12		0.01		0.22	
48. I would become immobile if I found myself on a turbulent aircraft.	**0.93**	0.04	0.00	0.13		0.11		0.02	
58. I refrain from handling snakes or spiders.	**0.74**	0.01	0.14	0.14		0.18		0.14	
6. At times, I experience feelings of sadness without any apparent cause.	0.12	**0.53**	0.12	0.01	0.16		0.12		
43. I frequently experience concern about disappointing others.	0.20	**0.57**	0.07	0.01		0.10		0.17	
49. I find that my behavior is readily disrupted.	0.05	**0.93**	0.06	0.01		0.17		0.14	
50. It’s challenging for me to remove certain thoughts from my mind.	0.11	**0.87**	0.01	0.00		0.06		0.09	
57. I frequently wake up with numerous thoughts circulating in my mind.	0.04	**0.87**	0.14	0.20		0.08		0.02	
14. I frequently experiment with new activities to gauge my enjoyment of them.	0.00	0.18	**0.98**	0.18		0.09		0.16	
18. I tend to become engrossed in new projects.	0.25	0.20	**0.99**	0.21		0.16		0.07	
32. I am continually discovering new and intriguing activities to pursue.	0.14	0.07	**0.84**	0.20		0.14		0.08	
12. I am driven to achieve success in my personal life.	0.10	0.01	0.06	**0.98**		0.22		0.02	
41. I exhibit a high level of persistence in pursuing my goals.	0.09	0.10	0.07	**0.91**		0.21		0.18	
65. I am proactive in devising plans to achieve my life goals.	0.03	0.11	0.01	**0.85**		0.21		0.14	
16. Positive news brings me immense joy.	0.14	0.17	0.02	0.18		**0.83**		0.12	
25. I experience a unique thrill when I receive praise for my accomplishments.	0.22	0.16	0.11	0.10		**0.86**		0.02	
36. I consistently celebrate whenever I achieve something significant.	0.17	0.02	0.01	0.08		**0.85**		0.01	
27. There are occasions when I find it challenging to refrain from speaking, even when I realize I should remain silent.	0.16	0.14	0.22	0.05		0.02		**0.78**	
28. I frequently engage in risky behaviors without considering the potential consequences.	0.10	0.24	0.21	0.04		0.05		**0.96**	
38. I often engage in impromptu actions.	0.01	0.03	0.08	0.05		0.03		**0.45**	

Note: FFFS: Flight Fight-Freeze System, BIS: Behavioral Inhibition System, RI: Reward Interest, GDP: Goal-Drive Persistence, RR: Reward Reactivity, IMP: Impulsivity

The first factor, “Flight Fight-Freeze System”, consisting of 5 items, accounted for 17.2% of the variance (eigenvalue = 3.77). The second factor, “Behavioral Inhibition System”, composed of 5 items, explained 13.8% of the total variance (eigenvalue = 3.02). The third factor, “Reward Interest”, composed of 3 items, explained 12.1% of the total variance (eigenvalue = 2.66). The fourth factor, “Goal-Drive Persistence”, composed of 3 items, explained 11.5% of the total variance (eigenvalue = 2.53). The fifth factor, “Reward Reactivity”, composed of 3 items, explained 9.9% of the total variance (eigenvalue = 2.17). The sixth factor, “Impulsivity”, composed of 3 items, explained 8% of the total variance (eigenvalue = 1.75).

Additionally, to confirm the factors extracted from the exploratory factor analysis, parallel analysis was also used, the results of which are shown in Fig. 1. The actual eigenvalues (blue line) represent the eigenvalues calculated from the real data of the RST-PQ-S scale. The random eigenvalues (red line) indicate the average eigenvalues obtained from 100 iterations of random simulations. These random eigenvalues were used as a benchmark to determine the number of significant factors. Figure 1 shows that the actual eigenvalues for six factors are significantly higher than the random eigenvalues, clearly confirming that both the actual analysis and the random simulation identified six factors as significant.

### Confirmatory Factor Analysis

**Fig. 2:**
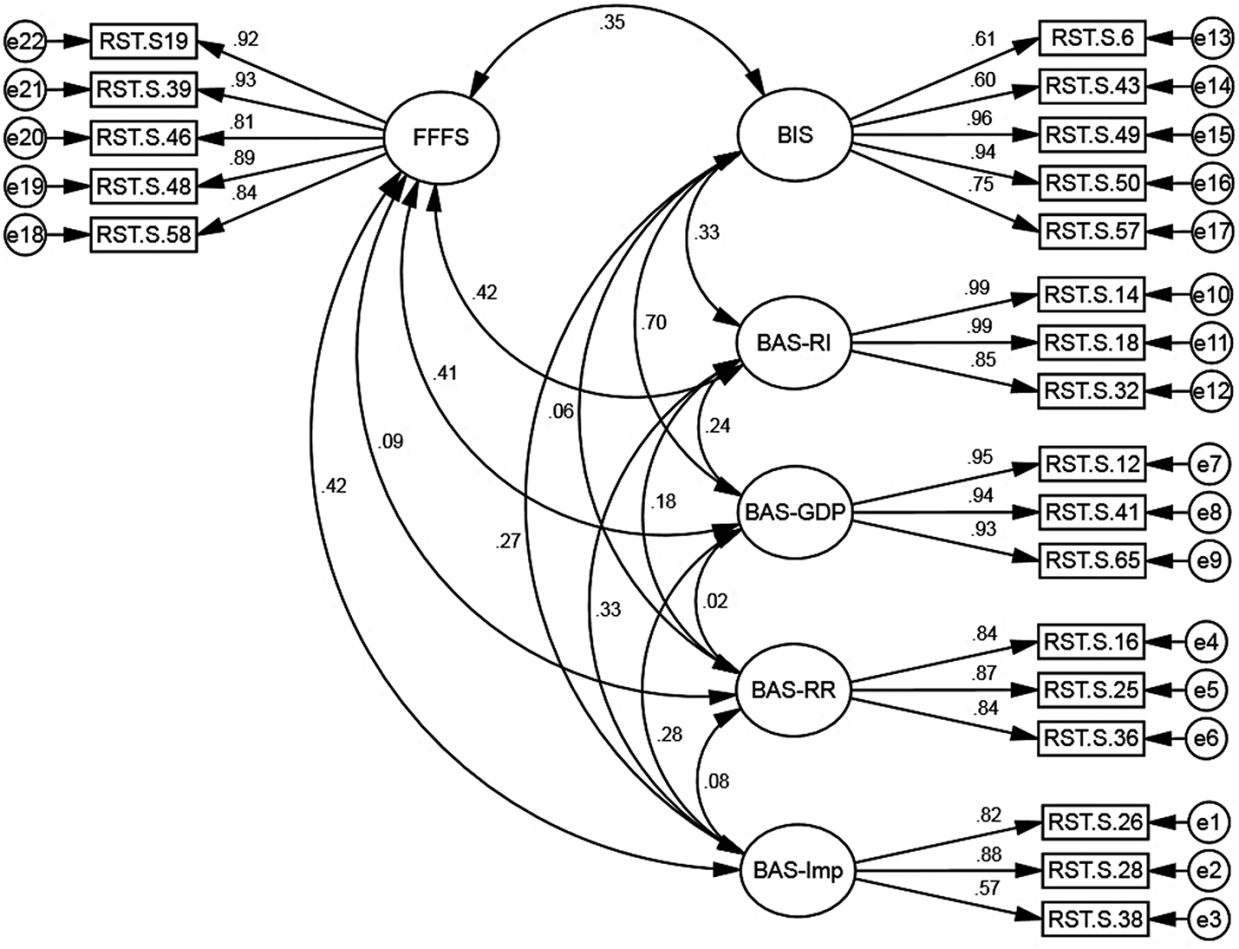
Structure of RST-PQ-S: First-order six-factor model of confirmatory factor analysis

The confirmatory factor analysis results indicate that the first-order six-factor model of RST-PQ-S demonstrates a very good fit to the data (Table 2). Subsequently, a second-order confirmatory factor analysis was conducted, whereby all six factors were loaded onto one higher-order construct. However, as the results show, the second-order confirmatory factor analysis model of RST-PQ-S demonstrates a poor fit to the data. Furthermore, the factor loadings for all 22 items of the first-order six-factor model of RST-PQ-S were higher than 0.40 and significant (Fig. 2).

**Table 2:** Fit indices of the four proposed models

MODELS	Χ^2^	Df	Χ^2^/df	CFI	RMSEA	GFI	AGFI	SRMR	AIC
Six-factor model	578.2	194	2.98	0.918	0.075	0.943	0.920	0.048	792.11
Second order	1110.5	203	5.47	0.889	0.113	0.795	0.751	0.087	1355.23

Χ²: Chi-square, Df: Degrees of freedom, Χ²/df: Chi-square to degrees of freedom ratio, CFI: Comparative Fit Index, RMSEA: Root Mean Square Error of Approximation, GFI: Goodness of Fit Index, AGFI: Adjusted Goodness of Fit Index, SRMR: Standardized Root Mean Square Residual, AIC: Akaike Information Criterion

### Gender Comparison

The findings from the independent t-test revealed non-significant differences between women and men across the six subscales of the RST-PQ-S (*p* > 0.05).

### Convergent and Discriminant Validity

To assess convergent validity, the Fornell and Larcker criterion and Pearson correlation were used. Given that the AVE and CR values for the six factors of RST-PQ-S were above 0.50 and 0.70, respectively, and that the CR values were greater than AVE for all factors, the scale demonstrates acceptable convergent validity based on the Fornell and Larcker criterion (Table 3). Additionally, the relationship between the six factors RST-PQ-S and the five factors NEO-FFI is shown in Table 4. Positive and significant relationships were found between RI, GDP, and RR scales with extraversion and agreeableness. Furthermore, a positive and significant relationship was observed between BAS-Imp and extraversion and neuroticism, while a negative and significant relationship was found with conscientiousness and agreeableness. Moreover, BIS showed a negative and significant relationship with conscientiousness and extraversion and a positive and significant relationship with openness and neuroticism. Additionally, FFS exhibited a positive and significant relationship with conscientiousness and neuroticism and a negative and significant relationship with extraversion. Additionally, BIS and FFFS had significant negative and positive correlations with Positive Mental Health and Psychological Distress, respectively. Furthermore, BAS-RI and BAS-GDP showed significant positive and negative correlations with Positive Mental Health and Psychological Distress, respectively. BAS-RR also had a significant positive correlation with Positive Mental Health. Therefore, the results of the Pearson correlation test indicate that the RST-PQ-S scale has acceptable convergent and divergent validity (Table 4).

**Table 3:** Internal homogeneity of the factors

	1	2	3.1	3.2	3.3	3.4
1. FFFS:		0.361	0.113	0.081	0.141	0.196
2. BIS:		-	0.150	0.199	0.062	0.134
3. BAS						
3.1.Reward Interest			-	0.438	0.401	0.308
3.2.Goal-Drive persistence				-	0.541	0.444
3.3.Reward Reactivity					-	0.739
3.4.Impulsivity						-
Alpha	0.922	0.934	0.895	0.911	0.905	0.840
McDonald’s omega	0.920	0.931	0.890	0.908	0.902	0.838
Split-half	0.936	0.947	0.910	0.919	0.914	0.859
ICC	0.840	0.925	0.872	0.856	0.905	0.948
AVE	0.789	0.609	0.791	0.883	0.722	0.590
CR	0.949	0.881	0.918	0.957	0.886	0.807

Note. ICC = interclass correlation coefficient

**Table 4:** Correlation between RST-PQ-S scale and NEO-FFI

	RST-PQ-S Scale					
	BIS	FFFS	BAS-RI	BAS-GDP	BAS-RR	BAS-Imp
Five-Factor Model:						
Openness	0.18**	− 0.01	0.29**	0.11*	0.20**	0.02
Conscientiousness	− 0.15*	0.18**	0.07	0.25**	0.19**	− 0.10*
Extraversion	− 0.45**	− 0.27**	0.40**	0.35**	0.42**	0.35**
Agreeableness	− 0.04	0.06	0.28**	0.19**	0.12**	− 0.47**
Neuroticism	0.30**	0.41**	− 0.31**	− 0.26**	0.04	0.28**
positive mental health	− 0.38**	− 0.28**	0.19**	0.24**	0.25**	0.06
psychological distress	0.41**	0.31**	− 0.16**	− 0.19**	− 0.08	0.07

*: Significant on 0.05 level, **: significant on 0.01 level

### Reliability

The values of the split-half, Cronbach’s alpha, and McDonald’s omega for the six RST-PQ-S factors were above 0.70. Therefore, the results indicate acceptable internal consistency of the RST-PQ-S. Additionally, the ICC values for the six factors of RST-PQ-S were above 0.80, indicating that the reliability and stability of this questionnaire are also confirmed (see Table 3). Also, in this study, the internal consistency of Openness, Conscientiousness, Extraversion, Agreeableness, and Neuroticism factors of the NEO-FFI scale was evaluated based on Cronbach’s alpha, and their values ​​were 0.756, 0.725, 0.832, 706, and 719, respectively.

### Exploratory Graph Analysis

**Fig. 3:**
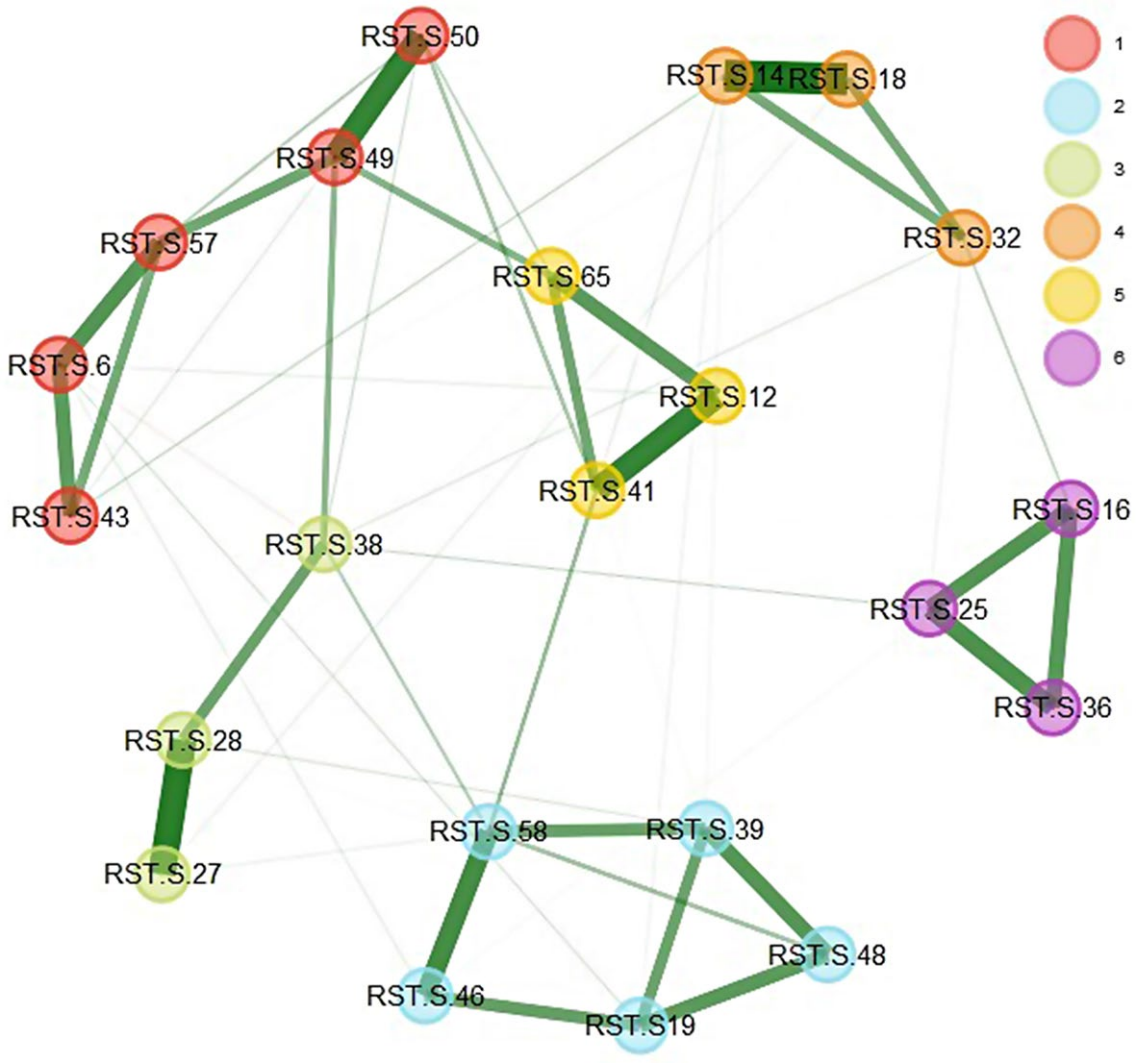
Dimensionality Results for EGA for the RST-PQ-S

The EGA identified a six-dimensional structure for the RST-PQ-S (Fig. 3). As shown in Fig. 3, all item dimensions align closely with the findings from the EFA. The analysis utilized a Glasso model combined with the walktrap algorithm, with the leading eigenvalue method applied for unidimensionality assessment.

To assess the stability of the dimensionality, a bootstrap analysis was conducted using 1,000 bootstrap samples. The results indicate that the median number of dimensions across bootstrap samples was six, with a standard error of approximately 0.063. The 95% confidence interval for the number of dimensions ranged from 5.88 to 6.12. Notably, all bootstrap samples consistently supported a six-dimensional structure.

According to the frequency analysis, a three-dimensional structure was observed in 99.6% of the 1,000 bootstrapped samples. This configuration was observed 996 times, indicating that the six-dimensional solution appears more stable and dominant across the bootstrapped samples.

**Fig. 4:**
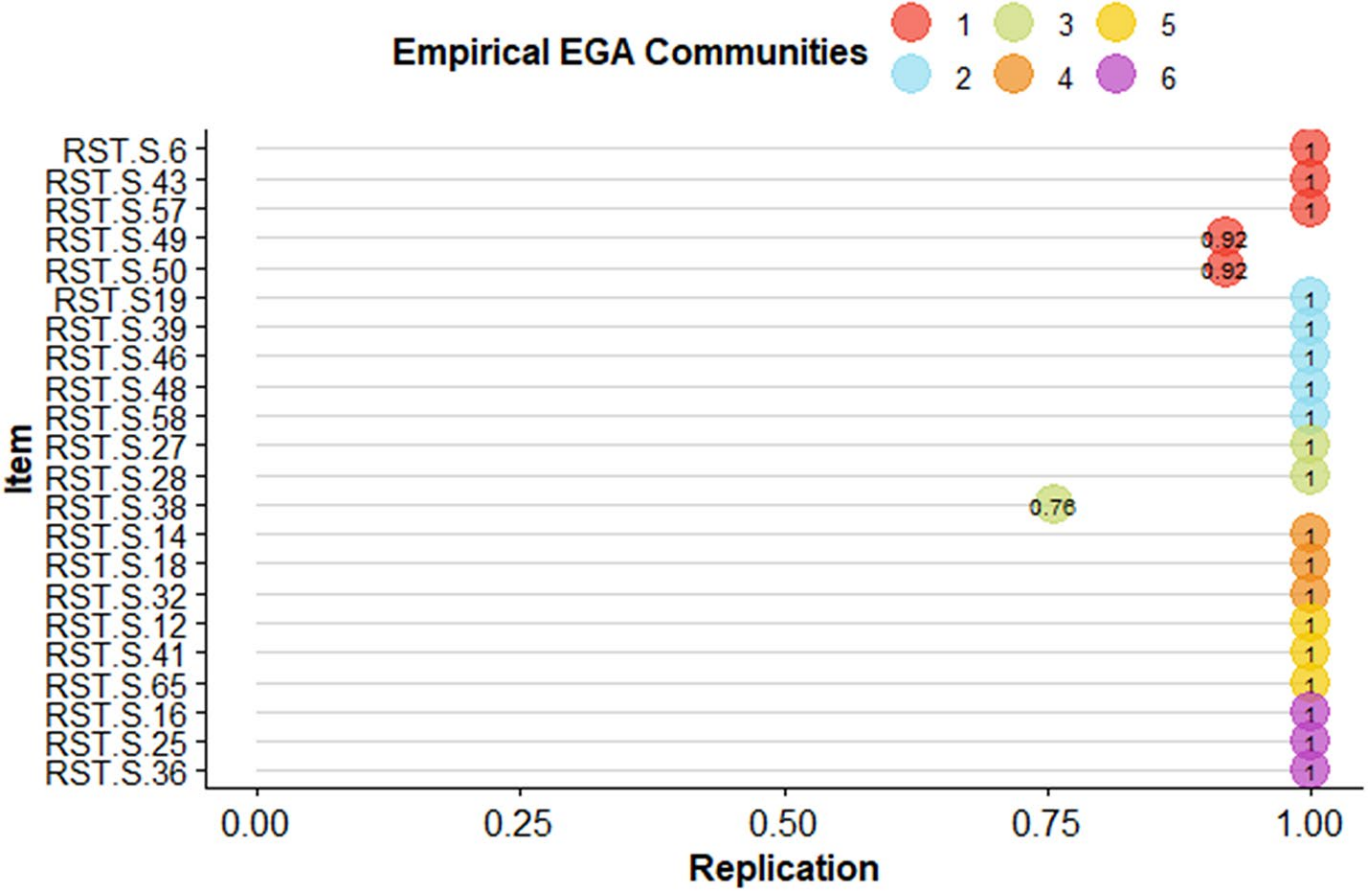
depicts the probability distribution of each symptom’s association with the community in which it was first identified by EGA. This determination was achieved through Bootstrap analysis, consisting of 10,000 iterations

Ultimately, Fig. 4 demonstrated the likelihood of each symptom maintaining its link with the initially recognized community throughout the bootstrap analysis, which underwent 1,000 iterations. The findings in Fig. 4 indicate that nearly all the items in the second dimension, barring item 38, displayed over 0.92 of the replications, potentially elucidating the remarkable structural coherence observed in this dimension. In contrast, the first dimension (BIS) exhibited fewer replications than the others.

## Discussion

The primary objective of this study was to translate and assess the psychometric properties of a RST-PQ-S within the Arabic-speaking population. To achieve this, we employed a range of statistical analyses to provide a comprehensive evaluation of the Arabic adaptation of the RST-PQ-S. Firstly, confirmatory factor analysis conducted on a subsample of participants revealed that the six-factor structure was the most fitting model for this context. The structure included the BIS, the FFFS, and four components of the BAS: reward reactivity, impulsivity, reward interest, and goal-drive persistence. The factor analysis revealed six distinct factors: “FFS” (5 items), which explained 17.2% of the variance; “BIS” (5 items), accounting for 13.8% of the variance; “reward interest” (3 items), explaining 12.1% of the variance; “goal-drive persistence” (3 items), contributing 11.5% of the variance; “reward reactivity” (3 items), explaining 9.9% of the variance; and “impulsivity” (3 items), accounting for 8% of the variance.

These results are consistent with the findings of the original study of the 22-item RST-PQ-S. The original study by Vecchione and Corr found that the RST-PQ-S also had six similar factors with comparable items [22]. This factor structure, albeit with a larger set of items, exhibits analogous psychometric properties when compared to the complete version in different countries and forms [15, 18, 49]. The CFA results from the current study showed strong factor loadings across all constructs of the RST-PQ-S. For instance, the loadings for the Flight-Fight-Freeze System range from 0.85 to 0.91, and for the Behavioral Inhibition System, the loadings are between 0.57 and 0.97. Similarly, high loadings are observed for Reward Interest (0.82 to 0.99), Goal-Drive Persistence (0.93 to 0.95), Reward Reactivity (0.84 to 0.87), and Impulsivity (0.57 to 0.88). In comparison, the Kordbagheri study also reported substantial factor loadings but with slightly lower ranges. For example, the loadings for Reward Interest items in Kordbagheri’s (2024) CFA range from 0.402 to 0.517, and for Goal-Drive Persistence, they range from 0.412 to 0.620. The Reward Reactivity items in Kordbagheri’s study had loadings between 0.431 and 0.699, and Impulsivity items ranged from 0.554 to 0.768. Overall, the current study’s CFA exhibits higher factor loadings, indicating a strong representation of the constructs, reinforcing the robustness, and validity of the RST-PQ-S.

Secondly, despite employing EGA as a dimensional approach, the network structure of RST-PQ-S included factors similar to those of the CFA-derived items. Since the current study was the first to utilize a network-based approach in the context of RST-PQ-S, it is impossible to compare the EGA results with previous studies directly. Third, the Cronbach’s alpha and split-half values for the six RST-PQ-S factors were above 0.70. Therefore, the results indicate acceptable internal consistency of the RST-PQ-S [22]. In the original study of RST-PQ-S, the authors found that the omega coefficients for all six subscales fell in the range of 0.60 to 0.70.

The current study found that different dimensions of the RST-PQ scale were significantly associated with mental health outcomes. Specifically, the BIS and the FFFS showed significant negative correlations with Positive Mental Health, with coefficients of − 0.38 and − 0.28, respectively. Conversely, these dimensions were positively correlated with psychological distress as measured by the K10, with correlations of 0.41 for BIS and 0.31 for FFFS. On the other hand, various components of the BAS displayed positive associations with PMH and negative associations with psychological distress. The BAS-RI, BAS-GDP, and BAS-RR were positively correlated with PMH (0.19, 0.24, and 0.25, respectively), while showing modest negative correlations with psychological distress (-0.16 for BAS-RI and − 0.19 for BAS-GDP). Interestingly, BAS-Imp did not show significant correlations with either PMH or psychological distress.

The findings suggest that higher activation of BIS and FFFS might contribute to lower positive mental health and greater psychological distress, which aligns with the theoretical understanding of these systems’ roles in sensitivity to punishment and threat. In contrast, the BAS components, particularly BAS-RI, BAS-GDP, and BAS-RR, appear to be protective factors for mental health, potentially enhancing positive mental states and reducing psychological distress through their roles in reward processing and goal-directed behavior. Notably, similar studies examining these specific relationships between RST dimensions and mental health outcomes could not be found, indicating a gap in the literature. This study provides a novel insight into how RST may be applied in the context of mental health, offering a promising avenue for future research to further explore and validate these associations.

In the context of validity, AVE values for the six factors RST-PQ-S were higher than 0.5. The AVE measures the variance captured by a latent construct relative to the amount of variance due to measurement error. An AVE value higher than 0.50 indicates that the latent construct explains more than half of the variance in the observed variables, suggesting good convergent validity [50].

We evaluated the convergent validity by examining the relationship between the six factors RST-PQ-S and the five factors NEO-FFI. The significant correlations observed between the BIS and various personality traits provide implications for understanding individual differences in personality and behavior. The positive correlation between BIS and openness to new experiences aligns with the notion that individuals high in BIS sensitivity may exhibit heightened responsiveness to novelty and a propensity for exploring new ideas and experiences. On the other hand, the negative correlation between BIS and conscientiousness suggests that individuals with elevated BIS scores may demonstrate lower levels of self-discipline, organization, and goal-directed behavior. Similarly, the negative correlation with extraversion implies that those with higher BIS sensitivity may be more reserved, withdrawn, and less inclined toward social engagement.Furthermore, the positive correlation between BIS and neuroticism underscores the association between BIS sensitivity, heightened emotional reactivity, and susceptibility to experiencing negative affect [15]. These findings contribute to a deeper understanding of how individual differences in BIS sensitivity relate to various aspects of personality functioning and may have implications for predicting behavior across different contexts, such as decision-making, risk-taking, and coping strategies [7]. The observed correlations between the FFFS and the Big Five personality traits were evaluated. According to Gray’s Reinforcement Sensitivity Theory, FFFS represents a motivational system geared toward detecting and responding to potential environmental threats (1). The negative correlation between FFFS and extraversion suggests that individuals with higher FFFS sensitivity may tend towards introversion and avoidance of highly stimulating or arousing social situations. Conversely, the positive correlation with conscientiousness indicates that individuals with elevated FFFS activation may demonstrate greater self-control, organization, and planning, possibly to manage potential threats. The absence of significant correlations with openness to new experiences and agreeableness suggests that FFFS sensitivity may not directly relate to individual differences in intellectual curiosity or interpersonal warmth but rather reflects a distinct aspect of personality related to threat sensitivity and approach-avoidance behavior. These results highlight the significance of considering neurobiological and personality aspects when examining individual variations in stress response mechanisms and their consequences for behavior and adjustment in demanding contexts.

While the findings are promising, several limitations must be acknowledged. Firstly, the study did not assess face and content validity. Secondly, there is a scarcity of studies using EGA to evaluate the psychometric properties of scales within the behavioral sciences. Thirdly, the cross-sectional design of this study prevents the establishment of causal relationships. Fourthly, the Arabic versions of the positive mental health and psychological distress scales were only tested in a sample of university students and staff. Additionally, the study’s focus on a university setting may limit the generalizability of the results. Future research should explore the criterion validity of the short form across diverse outcome measures and within larger, more representative samples. Furthermore, incorporating other network analysis tools in future studies could improve the predictability of symptoms.

### Practical Implications

The successful validation of the RST-PQ-S in the Arabic-speaking population provides clinicians with a robust tool for assessing personality traits closely associated with anxiety disorders. By understanding the relationship between traits such as behavioral inhibition, reward reactivity, and impulsivity, clinicians can better tailor treatment plans to individual patients. For example, individuals with high behavioral inhibition or impulsivity may benefit from interventions focused on reducing anxiety sensitivity and enhancing emotional regulation. Identifying these traits through the RST-PQ-S can also enable clinicians to track treatment progress and predict the likelihood of developing anxiety-related disorders, allowing for more proactive care.

The factor structure identified in this study further supports the notion that targeting specific personality dimensions—such as goal-drive persistence or reward interest—can enhance therapeutic outcomes. For instance, individuals with low reward interest or goal persistence may experience difficulties with motivation, which can intensify anxiety. By addressing these traits, clinicians can develop more personalized interventions that tackle the underlying causes of anxiety, ultimately improving treatment efficacy. From a research perspective, the Arabic version of the RST-PQ-S opens new opportunities for investigating the role of personality traits across various psychological conditions beyond anxiety, including depression, eating disorders, and substance use disorders. This validated tool offers researchers a reliable measure to examine how different personality dimensions interact with other psychological constructs. Moreover, the application of network analysis, as demonstrated in this study, can help researchers identify intricate relationships between personality traits and clinical symptoms, contributing to a deeper, more nuanced understanding of mental health. Additionally, the successful adaptation of the RST-PQ-S for Arabic-speaking populations underscores its potential for cross-cultural comparisons. By utilizing this version of the questionnaire in different cultural contexts, researchers can explore how reinforcement sensitivity theory manifests across diverse populations and how cultural factors shape the expression of anxiety and other emotional disorders. These insights can inform global mental health practices and lead to the development of culturally sensitive assessment tools and interventions that are more effective in addressing the needs of diverse populations.

## Conclusion

This study aimed to examine the factor structure of the Arabic version of the RST-PQ-S. Our findings validate the six-factor model, which was confirmed through both network analysis and traditional CFA, with both methods yielding consistent and satisfactory results. Additionally, we observed specific correlations between certain personality traits and the subscales of the RST-PQ-S. These results not only contribute to the psychometric validation of the tool in an Arabic-speaking context but also offer valuable insights for clinicians working with individuals suffering from anxiety disorders. By highlighting how specific personality traits are linked to anxiety, our findings can inform more targeted and effective interventions in clinical settings. Furthermore, this study paves the way for further research into the role of personality traits in mental health, particularly within Arabic-speaking populations. The validated Arabic version of the RST-PQ-S can serve as a reliable instrument for both clinical assessments and research, with potential applications extending to a broader range of psychological conditions.

## Data Availability

The corresponding author will provide the datasets used and/or analyzed during the current work upon reasonable request.

## References

[1] Corr PJ. Reinforcement sensitivity theory (RST). Introduction; 2008.

[2] Harmon-Jones E. Anger and the behavioral approach system. Pers Indiv Differ. 2003;35(5):995–1005.

[3] Hirsh JB, Kang SK. Mechanisms of identity conflict: Uncertainty, anxiety, and the behavioral inhibition system. Personality social Psychol Rev. 2016;20(3):223–44. 10.1177/1088868315589475 26048875

[4] Field AP. The behavioral inhibition system and the verbal information pathway to children’s fears. J Abnorm Psychol. 2006;115(4):742. 17100531 10.1037/0021-843X.115.4.742

[5] Corr PJ. Reinforcement sensitivity theory and personality. Neurosci biobehavioral reviews. 2004;28(3):317–32. 10.1016/j.neubiorev.2004.01.005 15225974

[6] Corr PJ. Reinforcement sensitivity theory of personality questionnaires: Structural survey with recommendations. Pers Indiv Differ. 2016;89:60–4.

[7] Smillie LD, Pickering AD, Jackson CJ. The new reinforcement sensitivity theory: Implications for personality measurement. Personality social Psychol Rev. 2006;10(4):320–35. 10.1207/s15327957pspr1004_3 17201591

[8] Gray J, McNaughton N. The psychology of Anxiety and Enquiry in to the functions of the septo hippocampus system. In: New York: Oxford University Press; 2000.

[9] Scholten MR, van Honk J, Aleman A, Kahn RS. Behavioral inhibition system (BIS), behavioral activation system (BAS) and schizophrenia: Relationship with psychopathology and physiology. J Psychiatr Res. 2006;40(7):638–45. 16643947 10.1016/j.jpsychires.2006.03.005

[10] Corr PJ, McNaughton N. Neuroscience and approach/avoidance personality traits: A two stage (valuation–motivation) approach. Neurosci biobehavioral reviews. 2012;36(10):2339–54. 10.1016/j.neubiorev.2012.09.013 23041073

[11] Corr PJ, Matthews G. The Cambridge handbook of personality psychology. Cambridge University Press; 2020.

[12] Corr PJ, Cooper AJ. The reinforcement sensitivity theory of personality questionnaire (RST-PQ): development and validation. Psychol Assess. 2016;28(11):1427. 26845224 10.1037/pas0000273

[13] Jackson CJ. Jackson-5 scales of revised Reinforcement Sensitivity Theory (r-RST) and their application to dysfunctional real world outcomes. J Res Pers. 2009;43(4):556–69.

[14] Amiri S, Nadilyu B, K., Ghasemzade M. (2019). Reliability, validity, and factor structure of the Persian version of the Children’s Reinforcement Sensitivity Scale. Iran J Psychiatry Clin Psychol.

[15] Kordbagheri A, Abdelrahman RM, Alsharif AF, Ahmed M. Psychometric properties of the Persian version of the Reinforcement Sensitivity Theory Personality Questionnaire (RST-PQ). Acta Psychol. 2024;244:104211. 10.1016/j.actpsy.2024.104211 38461582

[16] Eriksson LJK, Jansson B, Sundin Ö. Psychometric properties of a Swedish version of the reinforcement sensitivity theory of personality questionnaire. Nordic Psychol. 2019;71(2):134–45. 10.1080/19012276.2018.1516563.

[17] Pugnaghi G, Cooper A, Ettinger U, Corr PJ. The Psychometric Properties of the German Language Reinforcement Sensitivity Theory-Personality Questionnaire (RST-PQ). J Individual Differences. 2018a;39(3):182–90. 10.1027/1614-0001/a000262.

[18] Wytykowska A, Fajkowska M, Domaradzka E, Jankowski KS. Construct validity of the Polish version of the reinforcement sensitivity theory-personality questionnaire. Pers Indiv Differ. 2017;109:172–80. 10.1016/j.paid.2016.12.054.

[19] Floyd FJ, Widaman KF. Factor analysis in the development and refinement of clinical assessment instruments. Psychol Assess. 1995;7(3):286.

[20] Yang C, Nay S, Hoyle RH. Three Approaches to Using Lengthy Ordinal Scales in Structural Equation Models:Parceling, Latent Scoring, and Shortening Scales. Appl Psychol Meas. 2010;34(2):122–42. 10.1177/0146621609338592. 20514149 10.1177/0146621609338592 PMC2877522

[21] Moshagen M. The Model Size Effect in SEM: Inflated Goodness-of-Fit Statistics Are Due to the Size of the Covariance Matrix. Struct Equation Modeling: Multidisciplinary J. 2012;19(1):86–98. 10.1080/10705511.2012.634724.

[22] Vecchione M, Corr PJ. Development and validation of a short version of the Reinforcement Sensitivity Theory of Personality Questionnaire (RST-PQ-S). J Pers Assess. 2021;103(4):535–46. 32783537 10.1080/00223891.2020.1801702

[23] Kordbagheri A, Kordbagheri M, Tayim N, Fakhrou A, Davoudi M. Using advanced machine learning algorithms to predict academic major completion: A cross-sectional study. Comput Biol Med. 2025;184:109372. 39531920 10.1016/j.compbiomed.2024.109372

[24] Abdelrahman RM, Ahmed M, Tayim N, Kordbagheri M. Identification of the Core Characteristics of Vulnerable/Hypersensitive Narcissism and its Association with the Dark Triad in a Large International Sample: A Network Analysis Study. Psychiatr Q. 2024;95(3):415–31. 38985386 10.1007/s11126-024-10082-x

[25] Azadi F, Dayri S, Kordbagheri A, Kordbagheri M. Psychometric Properties of the Mindfulness in Teaching Scale in a Sample of Iranian Teachers: Insight from a Network Analysis Approach. Mindfulness. 2024;15(9):2277–90.

[26] Davoudi M, Sadoughi M, Pourshahbaz A, Dolatshahi B, Astaneh AN. Exploring the structure of the university-students obsessive–compulsive tendency scale in Iranian university students: a network analysis study. BMC Res Notes. 2023;16(1):193. 37661261 10.1186/s13104-023-06474-0 PMC10476335

[27] Hevey D. Network analysis: a brief overview and tutorial. Health Psychol Behav Med. 2018;6(1):301–28. 34040834 10.1080/21642850.2018.1521283 PMC8114409

[28] Vasquez MM, Hu C, Roe DJ, Chen Z, Halonen M, Guerra S. Least absolute shrinkage and selection operator type methods for the identification of serum biomarkers of overweight and obesity: simulation and application. BMC Med Res Methodol. 2016;16:1–19. 27842498 10.1186/s12874-016-0254-8 PMC5109787

[29] Azarmehr T, Kordbagheri A, Sadat E, Kordbagheri M. (2024). Psychometric Properties of the Persian Version of the Principal Instructional Leadership Scale for High School Principals (PILS): Translation, Psychometric Properties, and Network Analysis. Asia-Pacific Educ Researcher, 1–12.

[30] Golino HF, Epskamp S. (2017). Exploratory graph analysis: A new approach for estimating the number of dimensions in psychological research. PLoS ONE, 12(6), e0174035. 10.1371/journal.pone.0174035 PMC5465941 28594839

[31] Golino H, Shi D, Christensen AP, Garrido LE, Nieto MD, Sadana R, Thiyagarajan JA, Martinez-Molina A. Investigating the performance of exploratory graph analysis and traditional techniques to identify the number of latent factors: A simulation and tutorial. Psychol Methods. 2020;25(3):292. 32191105 10.1037/met0000255 PMC7244378

[32] Golino HF, Demetriou A. Estimating the dimensionality of intelligence like data using Exploratory Graph Analysis. Intelligence. 2017;62:54–70.

[33] Pedersen G, Normann-Eide E, Eikenaes IU, Kvarstein EH, Wilberg T. Psychometric evaluation of the Norwegian Toronto Alexithymia Scale (TAS-20) in a multisite clinical sample of patients with personality disorders and personality problems. J Clin Psychol. 2022;78(6):1118–36. 10.1002/jclp.23270. 34716595 10.1002/jclp.23270

[34] Macovei CM, Bumbuc Ș, Martinescu-Bădălan F. The role of personality traits in mediating the relation between fear of negative evaluation and social interaction anxiety. Front Psychol. 2023;14:1268052. 37928579 10.3389/fpsyg.2023.1268052 PMC10621049

[35] Dong J, Xiao T, Xu Q, Liang F, Gu S, Wang F, Huang JH. Anxious Personality Traits: Perspectives from Basic Emotions and Neurotransmitters. Brain Sci. 2022;12(9):1141. 10.3390/brainsci12091141. PMID: 36138877; PMCID: PMC9496710. 36138877 10.3390/brainsci12091141 PMC9496710

[36] Halfaker DA, Akeson ST, Hathcock DR, Mattson C, Wunderlich TL. (2011). 3 - Psychological Aspects of Pain. In T. A. Lennard, S. Walkowski, A. K. Singla, & D. G. Vivian, editors, Pain Procedures in Clinical Practice (Third Edition) (pp. 13–22). Hanley & Belfus. 10.1016/B978-1-4160-3779-8.10003-X

[37] Coelho O, Pires R, Ferreira AS, Gonçalves B, AlJassmi M, Stocker J. Arabic version of the personality inventory for the DSM-5 (PID-5) in a community sample of United Arab Emirates Nationals. Clin Pract Epidemiol Mental Health: CP EMH. 2020;16:180. 32874193 10.2174/1745017902016010180 PMC7431702

[38] Almubaddel A. Psychometric properties of a Saudi Arabian version of the Positive Mental Health (PMH) scale. Psicol Refl Crít. 2022;35:29. 10.1186/s41155-022-00232-0. 10.1186/s41155-022-00232-0 PMC9489822 36125579

[39] Easton SD, Safadi NS, Wang Y, et al. The Kessler psychological distress scale: translation and validation of an Arabic version. Health Qual Life Outcomes. 2017;15:215. 10.1186/s12955-017-0783-9. 29078774 10.1186/s12955-017-0783-9 PMC5658946

[40] Kordbagheri M, Kordbagheri A, Tayim N, Davoudi M. Evaluation of the psychometric indicators of the Comprehensive Autistic Trait Inventory in Iranian adults. Asian J Psychiatry. 2024;91:103885. 10.1016/j.ajp.2023.103885 38157634

[41] Rajaeiramsheh F, Rezaie Z, Davoudi M, Ahmadi SM, Qorbani-Vanajemi M. Psychometric properties of the Persian versions of acceptance and action diabetes questionnaire (AADQ) and the diabetes acceptance and action scale (DAAS), in Iranian youth with type 1 diabetes. J Diabetes Metabolic Disorders. 2021;20(1):655–63. 10.1007/s40200-021-00796-1. 10.1007/s40200-021-00796-1 PMC8212288 34178858

[42] Fabrigar LR, Wegener DT. Exploratory factor analysis. Oxford University Press; 2011.

[43] Mabel OA, Olayemi OS. A comparison of principal component analysis, maximum likelihood and the principal axis in factor analysis. Am J Math Stat. 2020;10(2):44–54.

[44] Beauducel A, Herzberg PY. On the performance of maximum likelihood versus means and variance adjusted weighted least squares estimation in CFA. Struct Equ Model. 2006;13(2):186–203.

[45] Harrington D. Confirmatory factor analysis. Oxford University Press; 2009.

[46] Shek DT, Yu L. Confirmatory factor analysis using AMOS: a demonstration. Int J Disabil Hum Dev. 2014;13(2):191–204.

[47] Golino H, Christensen AP, Garrido LE. Invited commentary: Exploratory graph analysis in context. Revista Psicologia: Teoria e Prática. 2022;24(3):ePTPPA14197–14197.

[48] Yan Z, Yao Y. Variable selection method for fault isolation using least absolute shrinkage and selection operator (LASSO). Chemometr Intell Lab Syst. 2015;146:136–46.

[49] Pugnaghi G, Cooper A, Ettinger U, Corr PJ. (2018). The psychometric properties of the German language reinforcement sensitivity theory-personality questionnaire (RST-PQ). J Individual Differences.

[50] dos Santos PM, Cirillo MÂ. Construction of the average variance extracted index for construct validation in structural equation models with adaptive regressions. Commun Statistics-Simulation Comput. 2023;52(4):1639–50.

